# Supplementary material for: Generating synthetic mixed-type longitudinal electronic health records for artificial intelligent applications
Source: NPJ Digit Med. 2023 May 27;6:98. doi: 10.1038/s41746-023-00834-7 (PMC10224668; doi:10.1038/s41746-023-00834-7)
Supplement: Supplementary file 1 — Supplementary Information [file 41746_2023_834_MOESM1_ESM.pdf]

Supplementary Information

Generating Synthetic Mixed-type Longitudinal Electronic Health Records for Artificial Intelligent Applications

Jin Li, Benjamin J. Cairns, Jingsong Li, and Tingting Zhu

SUPPLEMENTARY NOTE 1 - METHODOLOGY

Related work

Generative adversarial networks (GANs) have been used in EHR data synthesis, which can augment limited clinical data or even replace sensitive patient information. As longitudinal EHR data can capture patients' status over time, generative approaches for EHR data synthesis in the previous literature have been extended from static data to clinical timeseries generation. EHRs consist of a set of heterogeneous data types, such as continuous-valued and discrete-valued features. When generating continuous-valued timeseries such as heart rate and respiratory rate in the critical care database, models such as C-RNN-GAN [1], R(C)GAN [2] and TimeGAN [3] can be adopted. In order to model the temporal dynamics in the real-valued timeseries, recurrent neural networks (RNNs) such as long short-term memory (LSTM) are used as the generator and discriminator in their architectures. For synthesizing discrete-valued timeseries data such as diagnostic ICD-codes, GANs variants such as SynTEG [4], LS-EHR [5], and DualAAE [6] models are proposed. For example, SynTEG generates time-stamped clinical events across patients' multiple visits. Its amended version — LS-EHR [5] model enhances the longitudinal EHR data synthesis by overcoming the performance drift through feedback mechanisms (including condition fuzzing, regularization and rejection sampling).

As EHRs are an amalgamation of heterogeneous data types, previous work has demonstrated the importance of synthesizing mixed-type EHRs for various clinical applications. Several models have been proposed to generate static EHRs of mixed data types, such as discrete-valued medical concepts and continuous-valued measurements [7–9]. However, for synthesizing clinical timeseries, most of the proposed models have been capable of synthesizing only a single data type (either continuous or discrete-valued timeseries separately). Consequently, previous work has tended to ignore the inter-dependencies among different data types, as shown in Supplementary Figure 1. In contrast, our proposed model can simultaneously generate both continuous-valued and discrete-valued timeseries, while capturing the inter-dependencies between the mixed-type data.

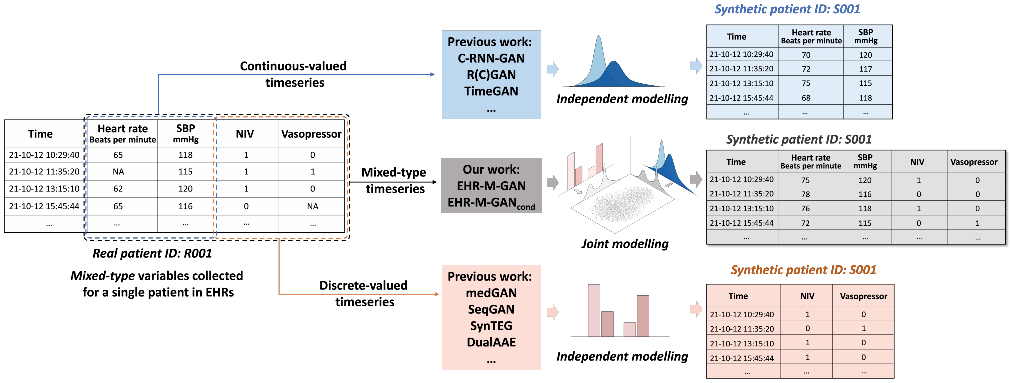

Supplementary Figure 1. Comparison of different generative models.

Implementation of GANs using LSTMs

GAN consists of two networks that are adversarially trained to compete against each other. The recurrent neural networks (RNNs) are instantiated considering simulating the temporal structure

for generating the sequential data. As shown in Fig. 1 (b. Network architecture) in the main article, the generator G accepts $\boldsymbol{v}_{1:T} \in \mathcal{T} \times \mathcal{V}$ as the input, which is a sequence of length T sampled independently from a prior distribution [2], such as Gaussian distribution or uniform distribution. In this study, uniform distribution on the unit interval is chosen as the prior for sampling the random noise. Then G is optimized to approximate the distribution of true data, $p_{\mathbf{x}}$, by generating samples $\hat{\mathbf{x}}_{1:T}$ that are hard for the discriminator to distinguish from. Meanwhile, the discriminator D is optimized to distinguish real samples $\mathbf{x}_{1:T}$ from synthetic samples $\hat{\mathbf{x}}_{1:T}$. Overall, the training of GAN is a minmax game with the following objective function:

$$\min_G \max_D V_{\text{GAN}} = \mathbb{E}_{\mathbf{x}\sim p_{\mathbf{x}}}[\log D(\mathbf{x})] + \mathbb{E}_{\boldsymbol{v}\sim p_{\boldsymbol{v}}}[\log(1 - D(G(\boldsymbol{v})))] \tag{1}$$

Conditional GAN is the extension case of GAN, where both the generator G and discriminator D receive conditional information $\mathbf{y} \in \mathcal{L} = \{1, 2, ..., |L|\}$ from $|L|$ classes [2]. In other words, the inputs are augmented by being concatenated with $\mathbf{y}$ at each timestamp, i.e., $\mathbf{x}_{1:T} \to [\mathbf{y}; \mathbf{x}_{1:T}]$. This formulation allows G to generate samples conditioned on the auxiliary information of $|L|$-dimensional categorical labels. In this case, the objective function becomes:

$$\begin{aligned}\min_G \max_D V_{\text{CGAN}} =& \mathbb{E}_{\mathbf{y},\mathbf{x}\sim p_{\mathbf{y},\mathbf{x}}}[\log D(\mathbf{x}|\mathbf{y})] \\ &+ \mathbb{E}_{\mathbf{y}\sim p_{\mathbf{y}},\boldsymbol{v}\sim p_{\boldsymbol{v}}}[\log(1 - D(G(\mathbf{y}, \boldsymbol{v})|\mathbf{y}))]\end{aligned} \tag{2}$$

Shared latent space learning using dual-VAE

As shown in Supplementary Figure 2, the shared latent space is learnt by a dual-VAE network, which contains a pair of encoders (parameterized as $\phi_{Enc^{\mathcal{C}}}$ and $\phi_{Enc^{\mathcal{D}}}$), and a pair of decoders (parameterized as $\psi_{Dec^{\mathcal{C}}}$ and $\psi_{Dec^{\mathcal{D}}}$) of VAE networks, one for each type of timeseries. We found VAE preferable to vanilla autoencoder in our case, considering that (1) the KL regularization in VAE strengthens the learning of the compressed latent representations, which further narrows the domain gap for mixed-type features [10]; (2) VAE can be easily extended to the conditional learning scenario in EHR-M-GAN$_{\texttt{cond}}$; (3) VAE as a generative model can produce the probability distribution function of the input data (as opposed to autoencoder, which learns the embedding as a fixed set of vectors [11]), therefore enhancing the generative capability of the proposed model. The encoders map the observations into the latent space with $Enc(\mathbf{x}) \triangleq q_\phi(\mathbf{z}|\mathbf{x})$, while the decoders further map the representations into the reconstructed input with $Dec(\mathbf{z}) \triangleq p_\psi(\mathbf{x}|\mathbf{z})$. During the implementation, we found that except for pretraining the dual-VAE, integrating the optimization for decoders during the joint training stage also benefits the generative model from learning improved representations in the shared latent space.

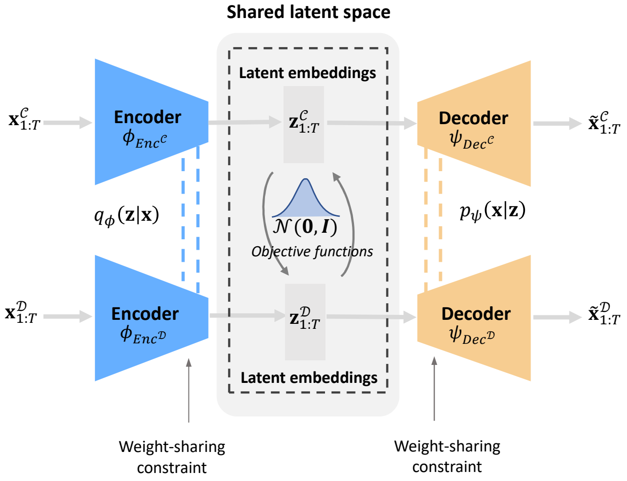

Supplementary Figure 2. The network architecture of dual-VAE during the pretraining stage.

In dual-VAE, we enforce a weight-sharing constraint [12] across certain layers within both the encoders pairs and decoders pairs to further eliminate the gap between domains (see Supplementary Figure 2). To be specific, only weights of the last few layers of the encoders and the

first few layers of the decoders are shared [13]. This forces the encoders to derive the same high-level representations while maintaining different low-level realizations. Meanwhile, it forces the decoders to share the same high-level semantics and decode them into different low-level feature space observations.

Comparison between LSTM and Bilateral-LSTM

To better compare with BLSTMs, we elaborate the architecture of the LSTM network. LSTM utilizes three gates to control the cell state in order to mitigate the problems of gradient vanishing and exploding that appears in the recurrent neural network (RNN) — an input gate $\mathbf{i}_t$ that controls the amount of input information to be passed along into the memory cell, a forget gate $\mathbf{f}_t$ which controls the amount of past information to be neglected, and an output gate $\mathbf{o}_t$ which controls the update of the new memory cell. The range of outputs from $\mathbf{i}_t$, $\mathbf{f}_t$ and $\mathbf{o}_t$ are limited by $[0, 1]$ due to the sigmoid activation function. At each time step t, the transition functions in LSTM are as follows:

$$\begin{aligned}
\mathbf{i}_t &= \sigma\left(\mathbf{W}_{iv}\boldsymbol{v}_t + \mathbf{W}_{ih}\mathbf{h}_{t-1} + \mathbf{b}_i\right) \\
\mathbf{f}_t &= \sigma\left(\mathbf{W}_{fv}\boldsymbol{v}_t + \mathbf{W}_{fh}\mathbf{h}_{t-1} + \mathbf{b}_f\right) \\
\mathbf{o}_t &= \sigma\left(\mathbf{W}_{ov}\boldsymbol{v}_t + \mathbf{W}_{oh}\mathbf{h}_{t-1} + \mathbf{b}_o\right) \\
\tilde{\mathbf{c}}_t &= \tanh\left(\mathbf{W}_{cv}\boldsymbol{v}_t + \mathbf{W}_{ch}\mathbf{h}_{t-1} + \mathbf{b}_c\right) \\
\mathbf{c}_t &= \mathbf{f}_t \odot \mathbf{c}_{t-1} + \mathbf{i}_t \odot \tilde{\mathbf{c}}_t \\
\mathbf{h}_t &= \mathbf{o}_t \odot \tanh\left(\mathbf{c}_t\right)
\end{aligned} \tag{3}$$

where $\mathbf{c}_t$ denotes the context vector, σ denotes the sigmoid activation function, and $\odot$ denotes the operation of element-wise multiplication.

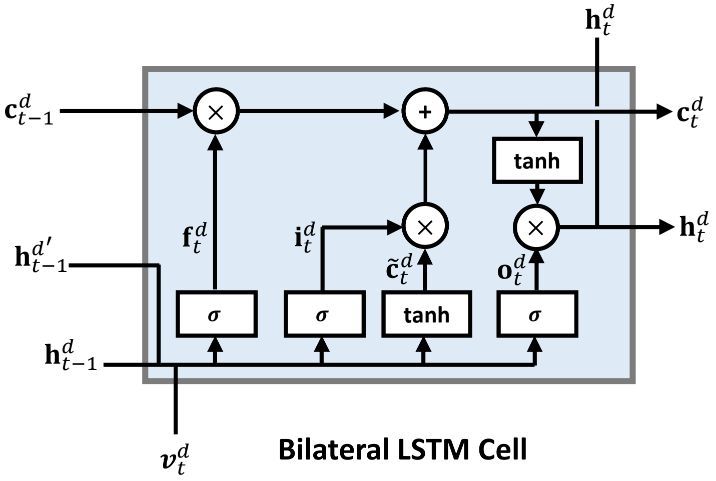

Supplementary Figure 3. Illustration of BLSTM cell.

Based on the basic structure of LSTM, the Bilateral Long Short-Term Memory (BLSTM) network is proposed (see Supplementary Figure 3). Equations that demonstrate the calculation of BLSTM units can be found in *Methodology* section in the main article.

Algorithm pseudocode

Algorithm 1. Algorithm of dual-VAE for pretraining.

Input: $\mathcal{D} = \{(\mathbf{x}_{i,1:T}^{\mathcal{C}}, \mathbf{x}_{i,1:T}^{\mathcal{D}})\}_{i=1}^{N}$, learning rate η_{VAE}, scalar loss weights β_0, β_1, β_2, β_3 (if `conditional`), minibatch size n_{mb}.

Initialize parameters: $\phi_{\text{Enc}}^{\mathcal{C}}, \phi_{\text{Enc}}^{\mathcal{D}}, \psi_{\text{Dec}}^{\mathcal{C}}, \psi_{\text{Dec}}^{\mathcal{D}}$

for number of pretrain iterations **do**

  Sample a minibatch of n_{mb} data samples: $\{(\mathbf{x}_{i,1:T}^{\mathcal{C}}, \mathbf{x}_{i,1:T}^{\mathcal{D}})\}_{i=1}^{n_{mb}} \overset{i.i.d.}{\sim} \mathcal{D}$

  // Map between features and latent representations:

  for $i = 1, 2, ..., n_{mb}, t = 1, 2, ..., T$ **do**

    $(\mathbf{z}_{i,t}^{\mathcal{C}}, \mathbf{z}_{i,t}^{\mathcal{D}}) = (Enc^{\mathcal{C}}(\mathbf{x}_{i,t}^{\mathcal{C}}, \mathbf{z}_{i,t-1}^{\mathcal{C}}), Enc^{\mathcal{D}}(\mathbf{x}_{i,t}^{\mathcal{D}}, \mathbf{z}_{i,t-1}^{\mathcal{D}}))$

    $(\tilde{\mathbf{x}}_{i,t}^{\mathcal{C}}, \tilde{\mathbf{x}}_{i,t}^{\mathcal{D}}) = (Dec^{\mathcal{C}}(\mathbf{z}_{i,t}^{\mathcal{C}}), Dec^{\mathcal{D}}(\mathbf{z}_{i,t}^{\mathcal{D}}))$

  // Estimate the loss terms:

  for $d \in \{\mathcal{C}, \mathcal{D}\}$ **do**

    $\mathcal{L}_d^{\text{ELBO}} = \frac{1}{n_{mb}} \sum_{i=1}^{n_{mb}} [-\mathbb{E}_{q_\phi(\mathbf{z}|\mathbf{x})}[\log p_\psi(\mathbf{x}|\mathbf{z})] + \beta_{\text{KL}} D_{\text{KL}}(q_\phi(\mathbf{z}|\mathbf{x}) \| p_\psi(\mathbf{z}))]$

    $\mathcal{L}^{\text{Match}} = \frac{1}{n_{mb}} \sum_{i=1}^{n_{mb}} [\mathbb{E}_{\mathbf{z} \sim p_{\mathbf{z}}} [\sum_{t \in \mathcal{T}} ||\mathbf{z}_t^{\mathcal{C}} - \mathbf{z}_t^{\mathcal{D}}||^2]]$

    $\mathcal{L}^{\text{Contra}} = \frac{1}{2n_{mb}} \sum_{i^d=1}^{n_{mb}} \sum_{i^{d'}=1}^{n_{mb}} [\mathcal{L}_{i^d, i^{d'}}^{\text{Contra}} + \mathcal{L}_{i^{d'}, i^d}^{\text{Contra}}]$

    $\mathcal{L}_d = \beta_0 \mathcal{L}_d^{\text{ELBO}} + \beta_1 \mathcal{L}^{\text{Match}} + \beta_2 \mathcal{L}^{\text{Contra}}$

    if `conditional`:

      $\mathcal{L}_d^{\text{Class}} = \frac{1}{n_{mb}} \sum_{i=1}^{n_{mb}} [\mathbb{E}_{\mathbf{z}^d \in \mathcal{H}^S} \text{CE}\left(f_{\texttt{linear}}^d(\mathbf{z}^d), \mathbf{y}\right)]$

      $\mathcal{L}_d = \beta_0 \mathcal{L}_d^{\text{ELBO}} + \beta_1 \mathcal{L}^{\text{Match}} + \beta_2 \mathcal{L}^{\text{Contra}} + \beta_3 \mathcal{L}_d^{\text{Class}}$

    // Update the network weights:

    $\phi_{\text{Enc}}^{\mathcal{C}} = \text{Adam}\left(\frac{\partial \mathcal{L}_{\text{VAE}}^{\mathcal{C}}}{\partial \phi_{\text{Enc}}^{\mathcal{C}}}, \eta_{\text{VAE}}\right), \psi_{\text{Dec}}^{\mathcal{C}} = \text{Adam}\left(\frac{\partial \mathcal{L}_{\text{VAE}}^{\mathcal{C}}}{\partial \psi_{\text{Dec}}^{\mathcal{C}}}, \eta_{\text{VAE}}\right)$

    $\phi_{\text{Enc}}^{\mathcal{D}} = \text{Adam}\left(\frac{\partial \mathcal{L}_{\text{VAE}}^{\mathcal{D}}}{\partial \phi_{\text{Enc}}^{\mathcal{D}}}, \eta_{\text{VAE}}\right), \psi_{\text{Dec}}^{\mathcal{D}} = \text{Adam}\left(\frac{\partial \mathcal{L}_{\text{VAE}}^{\mathcal{D}}}{\partial \psi_{\text{Dec}}^{\mathcal{D}}}, \eta_{\text{VAE}}\right)$

Return: $\psi_{\text{Dec}}^{\mathcal{C}}, \psi_{\text{Dec}}^{\mathcal{D}}$

Algorithm 2. Algorithm of EHR-M-GAN.

Input: $\mathcal{D} = \{(\mathbf{x}^{\mathcal{C}}_{i,1:T}, \mathbf{x}^{\mathcal{D}}_{i,1:T})\}_{i=1}^{N}$, pretrained decoder in dual-VAE for both domains $\psi^{\mathcal{C}}_{\text{Dec}}, \psi^{\mathcal{D}}_{\text{Dec}}$, learning rate η_{GAN}, minibatch size n_{mb}

Initialize parameters: $\theta^{\text{CRN}}_{G}, \mu^{\mathcal{C}}_{D}, \mu^{\mathcal{D}}_{D}$.

for number of training iterations **do**

    Sample a minibatch of n_{mb} random noise samples: $\{(\boldsymbol{v}^{\mathcal{C}}_{i,1:T}, \boldsymbol{v}^{\mathcal{D}}_{i,1:T})\}_{i=1}^{n_{mb}} \overset{i.i.d.}{\sim} \mathcal{V}$

    for $i = 1, 2, ..., n_{mb}, t = 1, 2, ..., T$ **do**

        // Generate synthetic latent codes using coupled-generator:

        $(\hat{\mathbf{z}}^{\mathcal{C}}_{i,t}, \hat{\mathbf{z}}^{\mathcal{D}}_{i,t}) = G^{\text{CRN}}((\boldsymbol{v}^{\mathcal{C}}_{i,t}, \boldsymbol{v}^{\mathcal{D}}_{i,t}), (\mathbf{h}^{\mathcal{C}}_{i,t-1}, \mathbf{h}^{\mathcal{D}}_{i,t-1}))$

        // Decode generated latent codes into observational space :

        $(\hat{\mathbf{x}}^{\mathcal{C}}_{i,t}, \hat{\mathbf{x}}^{\mathcal{D}}_{i,t}) = (Dec^{\mathcal{C}}(\hat{\mathbf{z}}^{\mathcal{C}}_{i,t}), Dec^{\mathcal{D}}(\hat{\mathbf{z}}^{\mathcal{D}}_{i,t}))$

    Sample a minibatch of n_{mb} real data samples: $\{(\mathbf{x}^{\mathcal{C}}_{i,1:T}, \mathbf{x}^{\mathcal{D}}_{i,1:T})\}_{i=1}^{n_{mb}} \overset{i.i.d.}{\sim} \mathcal{D}$, and a minibatch of n_{mb} synthetic data samples $\{(\hat{\mathbf{x}}^{\mathcal{C}}_{i,1:T}, \hat{\mathbf{x}}^{\mathcal{D}}_{i,1:T})\}_{i=1}^{n_{mb}} \overset{i.i.d.}{\sim} \mathcal{D}$

    // Distinguish real and fake samples using discriminators and estimate loss :

    $\mathcal{L}_{\text{GAN}} = \frac{1}{n_{mb}} \sum_{n=1}^{n_{mb}} [\log(D^{\mathcal{C}}(\mathbf{x}^{\mathcal{C}}_{i})) + \log(D^{\mathcal{D}}(\mathbf{x}^{\mathcal{D}}_{i}))] +$

        $[\log(1 - D^{\mathcal{C}}(\hat{\mathbf{x}}^{\mathcal{C}}_{i})) + \log(1 - D^{\mathcal{D}}(\hat{\mathbf{x}}^{\mathcal{D}}_{i})]$

    // Update network weights via Adam optimizer :

    $\theta^{\text{CRN}}_{G} = \text{Adam}\left(\frac{\partial \mathcal{L}_{\text{GAN}}}{\partial \theta^{\text{CRN}}_{G}}, \eta_{\text{GAN}}\right)$

    $\mu^{\mathcal{C}}_{D} = \text{Adam}\left(\frac{\partial \mathcal{L}_{\text{GAN}}}{\partial \mu^{\mathcal{C}}_{D}}, \eta_{\text{GAN}}\right), \;\; \mu^{\mathcal{D}}_{D} = \text{Adam}\left(\frac{\partial \mathcal{L}_{\text{GAN}}}{\partial \mu^{\mathcal{D}}_{D}}, \eta_{\text{GAN}}\right)$

// Synthesize M pairs of coupled mixed-types of features for M patients:

Sample $\{(\boldsymbol{v}^{\mathcal{C}}_{i,1:T}, \boldsymbol{v}^{\mathcal{D}}_{i,1:T})\}_{i=1}^{M} \overset{i.i.d.}{\sim} \mathcal{V}$

for $i = 1, 2, ..., M, t = 1, 2, ..., T$ **do**

    $(\hat{\mathbf{z}}^{\mathcal{C}}_{i,t}, \hat{\mathbf{z}}^{\mathcal{D}}_{i,t}) = G^{\text{CRN}}((\boldsymbol{v}^{\mathcal{C}}_{i,t}, \boldsymbol{v}^{\mathcal{D}}_{i,t}), (\mathbf{h}^{\mathcal{C}}_{i,t-1}, \mathbf{h}^{\mathcal{D}}_{i,t-1}))$

    $(\hat{\mathbf{x}}^{\mathcal{C}}_{i,t}, \hat{\mathbf{x}}^{\mathcal{D}}_{i,t}) = (Dec^{\mathcal{C}}(\hat{\mathbf{z}}^{\mathcal{C}}_{i,t}), Dec^{\mathcal{D}}(\hat{\mathbf{z}}^{\mathcal{D}}_{i,t}))$

Return: $\hat{\mathcal{D}} = \{(\hat{\mathbf{x}}^{\mathcal{C}}_{i,1:T}, \hat{\mathbf{x}}^{\mathcal{D}}_{i,1:T})\}_{i=1}^{M}$

SUPPLEMENTARY NOTE 2 - DATASETS

Preprocessing pipeline

We construct the pipeline of data preprocessing based on the work of MIMIC-Extract [14]. Three large-scale, publicly available datasets — MIMIC-III, eICU, and HiRID are processed based on the standard pipeline. The complete steps for data preprocessing include the following:

- Cohort selection: In cohort selection, patients in three ICU databases are selected based on the same predefined criteria.
- Timeseries features extraction: Then, the timeseries features are extracted based on the lists provided in Supplementary Table 1 - 6. Both continuous-valued and discrete-valued features are selected accordingly.
- Unit conversion and outlier filtering: Due to the fact that clinical data is often measured in different units, unit conversions are applied (such as converting Fahrenheit to Celsius for *Temperature*). For outlier filtering, a reasonable physiologically valid range is applied for different measurements.
- Semantic grouping: Next, semantically similar variables are grouped based on clinical concepts (such as *Heart Rate* is recorded as ItemID 211 in CareVUE EHR systems and ItemID 220045 under MetaVision EHR systems). A clinical taxonomy is used to aggregate features that are semantically equivalent [14].
- Hourly data aggregation: Following the preprocessing pipeline of MIMIC-EXTRACT, we aggregate the timeseries hourly further into a uniform bucket. The timeseries after the hourly aggregation for each patient, therefore, contain 24 values, each representing an hourly-aggregated timestamp.
- Imputation and normalization: Finally, the method of *Simple Imputation* [15] is used and normalization is applied to obtain the final result of the data matrix.

Cohort selection criteria

In line with the previous literature [14, 16], the cohort are selected based on the following criteria: (1) Only the first known ICU admission of the patient is selected. This is because patients who have multiple ICU admission records typically require specific treatments for life-support intervention; (2) the patient has to be an adult at the time of ICU admission (at least 15); (3) The duration of a patient's ICU stay is at least 12 hours and less than 10 days. This is because the treatment for patients who have long hours in the ICU stay usually indicates their physiological changes can not be directly linked to the positive effect of the treatment (but compensating for the life support treatment being taken off) [16].

Hourly data aggregation

This step generates hourly-resampled data. The temporal granularity, i.e., recording frequency for the raw timeseries data varies across different features. For example, in the HiRID dataset [17], physiological signals such as heart rate are frequently measured (e.g., most parameters under bedside monitoring are recorded every 2 minutes), while other features such as laboratory test results are measured infrequently (e.g. blood tests may be collected every few hours at most). In the MIMIC-III dataset, the nurse-verified vital signs are recorded approximately hourly (e.g., heart rate, blood pressure, respiratory rate) [18]. The detailed descriptions and statistical summaries for the original data for the three publicly available datasets — MIMIC-III [14, 18, 19], eICU [19, 20], and HiRID [17, 21], can be found in the corresponding papers in the literature.

Imputation method.

For continuous-valued timeseries, missing data is imputed based on the method of *Simple Imputation* [15]. The missing timeseries data is imputed as the last observed value, or individual-specific mean if no previous observation is provided. Else, if there is no observation for the subject, the imputation value is set to the global mean of the entire cohort. Compared to imputation methods developed upon customized RNN models or explicitly designed for the applied domains, it does not rely on additional information such as the prediction labels therefore more generalizable. Though simple, such a method has been widely applied in clinical timeseries analysis [22] including MIMIC-III datasets [14, 15, 23]. For discrete-valued timeseries, we followed the preprocessing

rules in MIMIC-EXTRACT. For intermittent interventions such as oral antibiotics, its status is regarded as 'not applied' when missing. For intervention with multi-hour continuous duration, such as mechanical ventilation, the missed status is considered to be consistent with the previous status until the new administration occurs. Therefore, the imputation method was not applied to the discrete-valued data.

Timeseries features extraction

Features of continuous-valued and discrete-valued timeseries are extracted for three critical care databases based on the following lists (for the MIMIC-III dataset, see Table S1, S2; for the eICU dataset see Table S3, S4; for the HiRID dataset, see Table S5, S6).

Supplementary Table 1. List of vital sign and laboratory test features for MIMIC-III dataset. Features are further extracted based on the preprocessed results of MIMIC-Extract (see Appendix A. Feature set in [14]). The dimension of continuous-valued features for the MIMIC-III dataset during the model's training is 78.

**Measurement**			
heart rate	respiratory rate	systolic blood pressure	diastolic blood pressure
mean blood pressure	oxygen saturation	temperature	glucose
central venous pressure	hematocrit	potassium	sodium
chloride	pulmonary artery pressure systolic	hemoglobin	ph
creatinine	blood urea nitrogen	bicarbonate	platelets
anion gap	co2 (etco2, pco2, etc.)	partial pressure of carbon dioxide	magnesium
white blood cell count	positive end-expiratory pressure set	calcium	fraction inspired oxygen set
red blood cell count	mean corpuscular hemoglobin concentration	mean corpuscular hemoglobin	mean corpuscular volume
tidal volume observed	partial thromboplastin time	prothrombin time inr	prothrombin time pt
phosphate	phosphorous	peak inspiratory pressure	calcium ionized
respiratory rate set	fraction inspired oxygen	tidal volume set	partial pressure of oxygen
cardiac index	co2	systemic vascular resistance	potassium serum
tidal volume spontaneous	plateau pressure	pulmonary artery pressure mean	cardiac output thermodilution
lactate	lactic acid	bilirubin	asparate aminotransferase
alanine aminotransferase	alkaline phosphate	positive end-expiratory pressure	albumin
troponin-t	neutrophils	lymphocytes	monocytes
ph urine	fibrinogen	lactate dehydrogenase	basophils
cardiac output fick	creatinine urine	pulmonary capillary wedge pressure	red blood cell count urine
white blood cell count urine	cholesterol	cholesterol hdl	post void residual
cholesterol ldl	chloride urine		

Supplementary Table 2. List of medical intervention features for MIMIC-III dataset, where **Features** indicates the name of the intervention features during model's training, **Category of treatment** shows the category of treatment that the specific intervention feature belongs to, and **Source** is the corresponding chart(s) where the variable is extracted based on[1]. The dimension of discrete-valued features for MIMIC-III dataset during model's training is 20.

Category of treatment	Features	Source
Oxygen therapy	supplemental oxygen<br>mechanical ventilation	chartevents, procedureevents_mv
Vasopressor	adenosine<br>dobutamine<br>dopamine<br>epinephrine<br>isuprel<br>milrinone<br>norepinephrine<br>phenylephrine<br>vasopressin	inputevents_cv, inputevents_mv
Antibiotics	antibiotics	prescriptions
Renal therapy	continuous renal replacement therapy	chartevents
Invasive lines	arterial line<br>central line	procedureevents_mv, chartevents
Colloid bolus	colloid bolus	inputevents_mv, inputevents_cv, chartevents
Crystalloid bolus	crystalloid bolus	inputevents_mv, inputevents_cv

[1]https://github.com/MIT-LCP/mimic-code

Supplementary Table 3. List of vital sign and laboratory test features for eICU dataset. Features are selected base on the recommendation from Rocheteau et al [24]. The dimension of continuous-valued features for eICU dataset during model's training is 55.

**Measurement**			
Hct	calcium	anion gap	MCH
troponin - I	MCHC	PT	PT - INR
-eos	potassium	-basos	albumin
-polys	lactate	glucose	creatinine
AST (SGOT)	Hgb	MPV	WBC × 1000
ALT (SGPT)	HCO3	MCV	-lymphs
Exhaled MV	RDW	chloride	sodium
bicarbonate	pH	urinary specific gravity	SaO2
Tidal Volume (set)	-monos	Heart Rate	BUN
platelets × 1000	total bilirubin	Exhaled TV (patient)	alkaline phos
Noninvasive BP Diastolic	Noninvasive BP Mean	Noninvasive BP Systolic	Base Excess
paO2	FiO2	Temperature	RBC
PTT	magnesium	RR	SpO2
total protein	paCO2	phosphate	

Supplementary Table 4. List of medical intervention features for eICU dataset, where **Features** indicates the name of the intervention features during model's training, **Category of treatment** shows the category of treatment that the specific intervention feature belongs to, and **Source** is the corresponding chart(s) where the variable is extracted based on[2]. The dimension of discrete-valued features for the eICU dataset during the model's training is 19.

Category of treatment	Features	Source
Oxygen therapy	supplemental oxygen<br>mechanical ventilation	`respiratorycharting, nursecharting, treatment`
Vasopressor	dopamine<br>epinephrine<br>norepinephrine<br>phenylephrine<br>vasopressin<br>milrinone<br>dobutamine	`infusionDrug`
Anesthesia	fentanyl<br>propofol<br>midazolam<br>dexmedetomidine	`infusionDrug`
Anticoagulants	heparin	`infusionDrug`
Insulin	insulin	`infusionDrug`
Antibiotics	antibiotics	`medication`

[2]https://github.com/MIT-LCP/eicu-code

Supplementary Table 5. List of vital sign and laboratory test features for HiRID dataset. Features are extracted based on the official HiRID preprocessing codes (meta-variables from `Merging stage` [3]) [17]. The dimension of continuous-valued features for HiRID dataset during model's training is 50.

**Measurement**			
HR	T Central	ABPs	ABPd
ABPm	NIBPs	NIBPd	NIBPm
PAPm	PAPs	PAPd	CO
SvO2(m)	ZVD	ST1	ST2
ST3	SpO2	ETCO2	RR
OUTurine/h	ICP	Liquor/h	a-BE
a_COHb	a_Hb	a_HCO3-	a_Lac
a_MetHb	a_pH	a_pCO2	a_PO2
a_SO2	K+	Na+	Cl-
Ca2+ ionizied	phosphate	Mg_lab	Urea
creatinine	INR	glucose	Hb
MCHC	MCV	platelet count	MCH
C-reactive protein	total white blood cell count		

[3] https://github.com/ratschlab/HIRID-ICU-Benchmark

Supplementary Table 6. List of medical intervention features for HiRID dataset, where **Features** indicates the name of the intervention features during model's training, **Category of treatment** shows the category of treatment that the specific intervention feature belongs to, and **Source** is the corresponding feature names in the official HiRID preprocessing codes (meta-variables from `Merging stage` [4]) [17] that we extracted based on. The dimension of discrete-valued features for HiRID dataset during model's training is 39.

Category of treatment	Features	Source
Oxygen therapy	supplemental oxygen	`vm23`
	mechanical ventilation	`vm60`
Crystalloids	crystalloids	`vm33`
Colloids	colloids	`vm34`
Renal therapy	haemofiltration	`vm72`
Blood transfusion	packed red blood cells	`pm35`
	FFP	`pm36`
	platelets	`pm37`
Vaspressor/inotropes	norepinephrine	`pm39`
	epinephrine	`pm40`
	dobutamine	`pm41`
	milrinone	`pm42`
	levosimendan	`pm43`
	theophyllin	`pm44`
	vasopressin	`pm45`
	desmopressin	`pm46`
Vasodilators	vasodilators	`pm47`
Antihypertensives	ACE inhibitors	`pm48`
	Calcium channel blockers	`pm50`
	Beta-blocker	`pm51`
Antiarrhythmics	adenosine	`pm53`
	digoxin	`pm54`
	amiodarone	`pm55`
	atropine	`pm56`
Antibiotics	antibiotics	`pm73`
	antimycotic	`pm74`
	antiviral	`pm75`
Insulin	insulin	`pm82, pm83`
Pain killers	opioid	`pm86`
	non-opioid	`pm87`
Steroids	steroids	`pm91`
Anticoagulants	heparin	`pm95`

[4] https://github.com/ratschlab/HIRID-ICU-Benchmark

SUPPLEMENTARY NOTE 3 - MODEL TRAINING

Implementation details

During the model training of EHR-M-GAN, the hyperparameters are optimized based on the comparison between the synthetic data and leave-out real data, estimated by mean maximum discrepancy (MMD) for continuous data and mean squared errors (MSEs) over the Bernoulli probability for discrete data, as the scoring functions. Visual inspection is also used during training to intuitively compare the resemblance between synthetic and real data. Supplementary Table 7 shows the hyperparameter values of the network architecture for searching over. The optimal hyperparameters for GANs' training is listed in our GitHub codebase (see *train_config.py* file). The model which generates the best results is saved and used for the final results.

Supplementary Table 7. List of hyperparameters of EHR-M-GAN.

**Hyperparameters**	**Searching space**
Batch size	{128, 256, 512}
Epochs for pretraining	{200, 500, 800}
Epochs for training GANs	{500, 800}
Rounds for jointly training $G/D/V$	{3/1/1, 5/1/1}
Learning rate for pretraining	{0.001, 0.0001, 0.0005}
Learning rate for training GANs	{0.001, 0.0001, 0.0005}
Depths for encoders and decoders	{3, 5}
Depths for generators	{3, 5}
Depths for discriminators	{1, 3, 5}
Sizes for encoders and decoders	{64, 128, 256}
Sizes for generators	{256, 512}
Sizes for discriminators	{256, 512}
Weight scalar for pretraining	{0.01, 0.1, 0.25, 0.5, 1, 2, 5}
Weight scalar for training GANs	{0.1, 0.5, 1, 5, 10, 20}
Optimizer	Adam

During the pretraining stage of the dual-VAE module, we implemented the VAEs with a recurrent neural network based on Google DeepMind's "DRAW" — Deep Recurrent Attentive Writer [25]. Instead of automatically generating the entire images/timeseries at once, it utilizes a sequential variational auto-encoding framework that enables the iterative generation of multivariate time-series. To monitor the training progress of the dual-VAE module, the reconstruction error between the original data (in the leave-out validation set) and its reconstructed version is calculated. For continuous data, the reconstruction error is calculated by *mean squared error (MSE)*. For the discrete-valued timeseries, the *error rate (1-Accuracy)* is implemented to test how well the discrete values in the EHR timeseries can be reconstructed. Additionally, randomly sampled patient trajectories from the reconstructed data and the corresponding original data are visualised and compared.

Furthermore, to stabilize GANs' training and overcome the problem of mode collapse, training strategies such as feature matching loss are utilized [26]. Feature matching is a regularizing objective that prevents the generator in GANs from overtraining on the current discriminator. It has been shown effective to stabilize the GANs' training as it calculates the *statistics* of the real data per minibatch, instead of directly maximizing the output of the discriminator. The formal definition of feature matching loss is described as follows:

$$L = \left\| \mathbb{E}_{x \sim p_{\text{data}}} \mathbf{f}(\mathbf{x}) - \mathbb{E}_{z \sim p_z(\mathbf{z})} \mathbf{f}(G(\mathbf{z})) \right\|_2^2$$

where $f(x)$ is the feature representation of the intermediate layer of the discriminator (layer before the final classification).

Ablation study for training dual-VAE

Multiple losses are placed when optimizing the shared latent space in the dual-VAE module. Except for the standard evidence lower bound (ELBO) loss in VAE, external losses, namely (1) Matching loss; (2) Contrastive loss, and (3) Semantic loss (for the conditional variation of our proposed model) are used. Also, during the implementation, the weight-sharing constraint is adopted for specific layers in dual-VAE's encoder and decoder pairs to extract the high-level representations from mixed-type inputs (see Section S.1.C *Shared latent space learning using dual-VAE* for details). In order to analyze the contribution of each aforementioned component when training dual-VAE, we perform an ablation study by varying the corresponding training configurations (see Supplementary Table 8) using the MIMIC-III dataset as an example. The performance for synthesizing continuous-valued timeseries is evaluated by maximum mean discrepancy (MMD) and discriminative score. For discrete-valued timeseries, the performance of GANs is evaluated by dimensional-wise probability (DWP) quantified by the averaged root mean squared errors (RMSEs) across all feature dimensions (see *Dimension-wise probability* section in the main text for details) and discriminative score. The results of the ablation study are shown in Table S11.

Supplementary Table 8. The ablation study for components in Dual-VAE on MIMIC-III dataset. 'Baseline' represents the proposed GAN models (EHR-M-GAN or EHR-M-GAN`cond`) with all components included. The quality of synthetic continuous-valued timeseries is evaluated by MMD and discriminative score (both the lower the better). The quality of synthetic discrete-valued timeseries is evaluated by averaged RMSEs in DWP and discriminative score (both the lower the better).

		Continuous-valued data		Discrete-valued data	
Model	Training configuration	MMD	Discriminative score	DWP (RMSEs)	Discriminative score
EHR-M-GAN	Baseline	**0.692 ± 0.034**	**0.746 ± 0.018**	**0.0104 ± 0.0006**	**0.813 ± 0.026**
	w/o Matching loss	0.722 ± 0.023	0.758 ± 0.015	0.0112 ± 0.0010	0.827 ± 0.019
	w/o Contrastive loss	0.719 ± 0.017	0.762 ± 0.012	0.0109 ± 0.0009	0.830 ± 0.023
	w/o Shared weights	0.704 ± 0.031	0.749 ± 0.019	0.0107 ± 0.0008	0.816 ± 0.035
EHR-M-GAN`cond`	Baseline	**0.604 ± 0.027**	**0.729 ± 0.025**	**0.0093 ± 0.0005**	**0.784 ± 0.024**
	w/o Matching loss	0.634 ± 0.026	0.736 ± 0.017	0.0106 ± 0.0013	0.795 ± 0.022
	w/o Contrastive loss	0.629 ± 0.022	0.739 ± 0.020	0.0108 ± 0.0007	0.796 ± 0.028
	w/o Semantic loss	0.647 ± 0.034	0.743 ± 0.011	0.0114 ± 0.0004	0.798 ± 0.030
	w/o Shared weights	0.609 ± 0.035	0.732 ± 0.014	0.0097 ± 0.0012	0.786 ± 0.027

As shown in Supplementary Table 8, both matching loss and contrastive loss contribute to the improvement of EHR-M-GAN's performance when generating mixed-type timeseries data. For example, the absence of the contrastive loss leads to a noticeable degradation in the quality of the synthetic discrete-valued timeseries (evaluated by discriminative score). Also, removing the matching loss causes the increase of the MMD between real and synthetic continuous-valued timeseries. The weight-sharing scheme between the encoder and decoder architectures in the dual-VAE also boosts GANs' performance but within a limited range. For EHR-M-GAN`cond` model, the effectiveness of the components that appear in EHR-M-GAN can still be observed. On the other hand, semantic loss, which injects conditional information into the networks, plays a major role in synthesizing more realistic patient trajectories. The results in Supplementary Table 8 show that the impact of the semantic loss exceeds the other two losses in learning the valid shared latent representations in dual-VAE.

Apart from being used for learning useful latent representations, VAEs alone is also a widely used generative model for synthesizing healthcare data [6, 27]. However, previous literature has found that a major drawback of VAEs is that it tends to produce "blurry" images and unrealistic samples [28–30], due to the pixel-/sample-wise reconstruction loss it relies on [30] (in contrast to adversarial loss in GANs). Therefore, we conduct the experiment on synthesizing

Supplementary Table 9. Performance comparison between dual-VAE and the proposed models evaluated by the discriminative score (the lower the better).

	**dual-VAE**	**EHR-M-GAN**	**EHR-M-GAN$_{cond}$**
Continuous-valued data	0.896 ± 0.030	0.746 ± 0.018	0.729 ± 0.025
Discrete-valued data	0.932 ± 0.036	0.813 ± 0.026	0.784 ± 0.024

mixed-type EHR timeseries on the MIMIC-III dataset with the proposed EHR-M-GAN and the dual-VAE module, serving as a non-GAN-based benchmark. The representativeness of the synthetic timeseries for both the continuous-valued and discrete-valued features is evaluated by the discriminative score. The results are shown in Supplementary Table 9. It can be seen that the proposed EHR-M-GAN produces more realistic synthetic samples than the dual-VAE module alone as the former produces a lower discriminative score.

SUPPLEMENTARY NOTE 4 - RESULTS

Dimension-wise probability

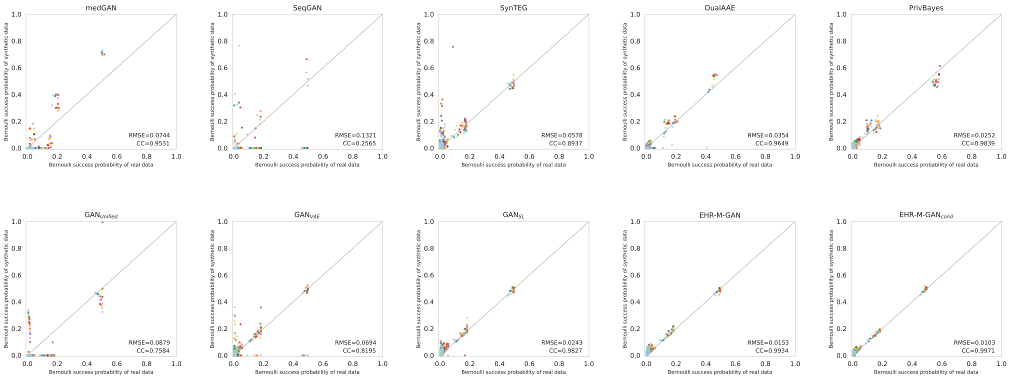

Supplementary Figure 4. Scatterplot of the dimension-wise probability test on the eICU dataset. The x-axis and y-axis represents the probability distribution for the real data and synthetic data with the same sample size, respectively. The optimal performance appears along the diagonal line. Each dot represents a treatment status at a particular time in the patient EHR data. The optimal performance appears along the diagonal line. The corresponding CCs ($[0, 1]$, the higher the better) and RMSEs ($[0, +\infty)$, the lower the better) are also calculated to quantify the probability distribution similarities between the real and synthetic EHRs timeseries.

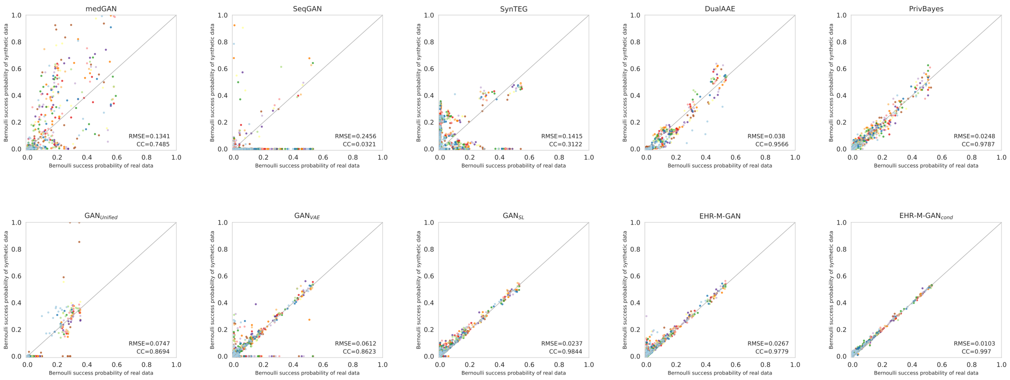

Supplementary Figure 5. Scatterplot of the dimension-wise probability test on the eICU dataset. The x-axis and y-axis represents the probability distribution for the real data and synthetic data with the same sample size, respectively. The optimal performance appears along the diagonal line. Each dot represents a treatment status at a particular time in the patient EHR data. The optimal performance appears along the diagonal line. The corresponding CCs ($[0, 1]$, the higher the better) and RMSEs ($[0, +\infty)$, the lower the better) are also calculated to quantify the probability distribution similarities between the real and synthetic EHRs timeseries.

Temporal characteristics

Supplementary Figure 6 - 8 show the autocorrelation function (ACF) of real timeseries and synthetic timeseries generated by EHR-M-GAN on continuous-valued features (including *Heart Rate*, *Oxygen Saturation*, *Respiratory Rate*, *Systolic Blood Pressure*, and *Temperature*) and discrete-valued features (including *Vasopressor* and *Mechanical Ventilation*). The averaged ACF is calculated over the population sampled randomly from both real and synthetic patient data. The averaged autocorrelation for real patient trajectories (solid blue line) and synthetic patient trajectories (red dashed line) are calculated, with the light-colored regions indicating the corresponding 95% confidence interval. The root-mean-square errors (RMSEs) are also calculated for the two curves on each variable to quantitatively evaluate the temporal characteristics captured by the synthetic data.

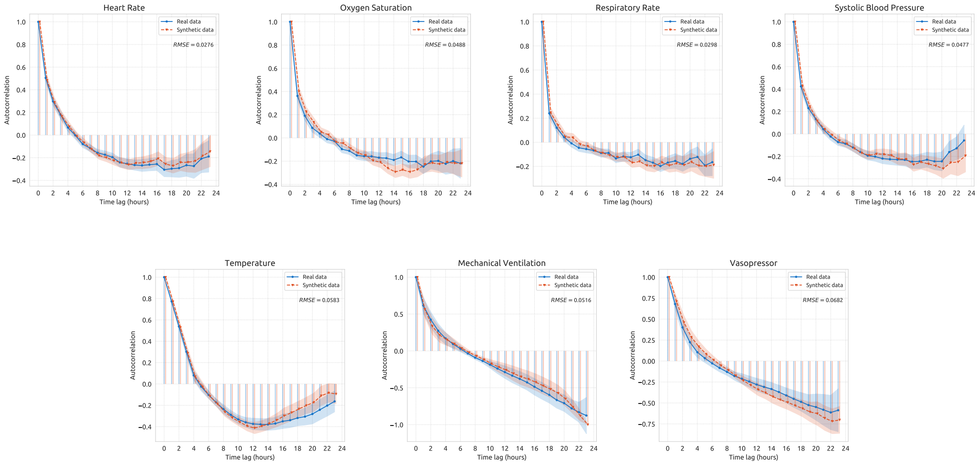

Supplementary Figure 6. Autocorrelation function (ACF) of real data and synthetic data generated by EHR-M-GAN on MIMIC-III dataset.

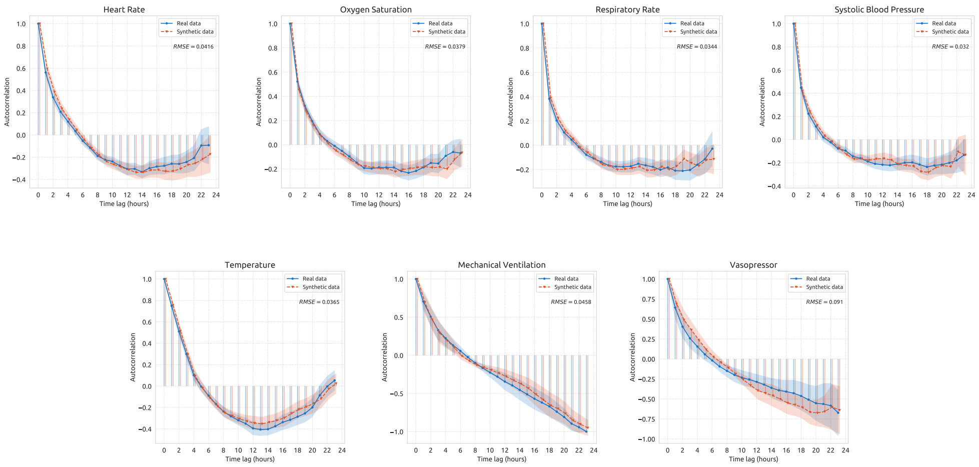

Supplementary Figure 7. **Autocorrelation function (ACF) of real data and synthetic data generated by EHR-M-GAN on eICU dataset.**

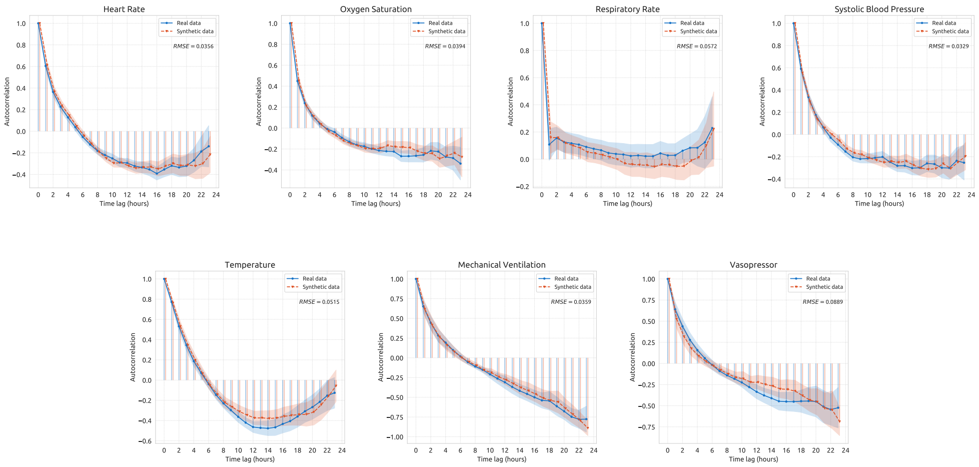

Supplementary Figure 8. **Autocorrelation function (ACF) of real data and synthetic data generated by EHR-M-GAN on HiRID dataset.**

Embedding visualisation

We apply t-SNE to qualitatively visualise the latent representations generated by EHR-M-GAN and EHR-M-GAN$_{\texttt{cond}}$ on three critical care databases. The latent embedding vectors are induced by the encoders in the *dual-VAE* during learning the shared latent space representations (See Methods section for details). The t-SNE embedding results on raw timeseries are also included for comparison.

It can be seen that better separability of the representation clusters in the shared latent space is shown in the embeddings obtained from EHR-M-GAN$_{\texttt{cond}}$ compared with EHR-M-GAN and raw data. This illustrates the superiority of the EHR-M-GAN$_{\texttt{cond}}$ in terms of learning the contextual information from the patient trajectories. It therefore can be inferred that the conditional extension of the proposed model can further yield benefits by synthesizing condition-specific EHR timeseries with respect to distinctive patient health status.

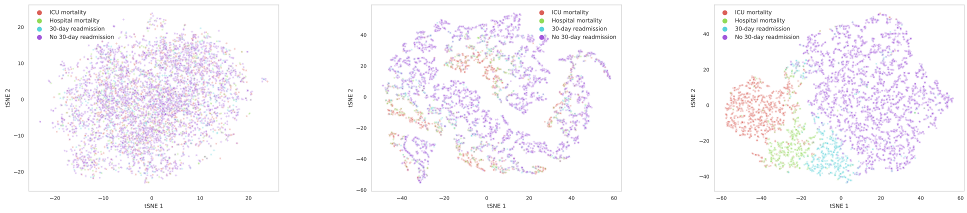

(a) (b) (c)

Supplementary Figure 9. t-SNE embedding visualization from MIMIC-III dataset on (a) raw patient trajectories, (b) latent embeddings generated with EHR-M-GAN, and (c) latent embeddings generated with EHR-M-GAN$_{\texttt{cond}}$.

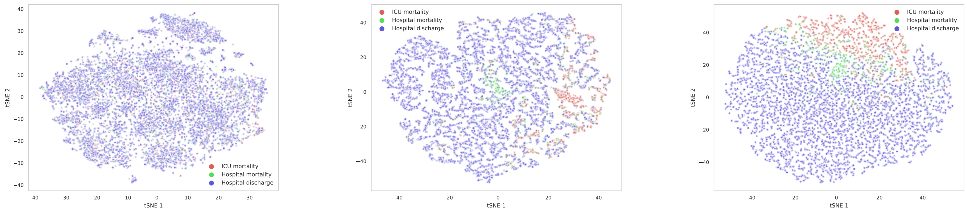

(a) (b) (c)

Supplementary Figure 10. t-SNE embedding visualization from eICU dataset on (a) raw patient trajectories, (b) latent embeddings generated with EHR-M-GAN, and (c) latent embeddings generated with EHR-M-GAN$_{\texttt{cond}}$.

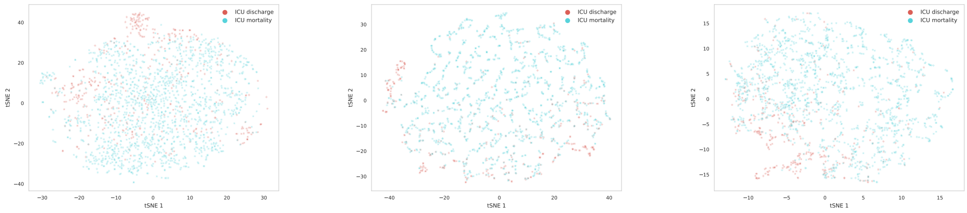

(a) (b) (c)

Supplementary Figure 11. t-SNE embedding visualization from HiRID dataset on (a) raw patient trajectories, (b) latent embeddings generated with EHR-M-GAN, and (c) latent embeddings generated with EHR-M-GAN$_{\texttt{cond}}$.

Patient trajectories visualisation

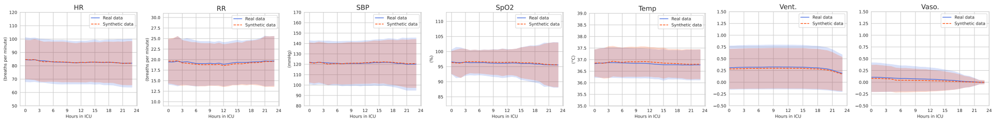

Supplementary Figure 12. Comparison of patient trajectories. The distribution of values at each timepoint (mean and standard deviation) are compared between real and synthetic patient trajectory produced by EHR-M-GAN$_{\texttt{cond}}$, under the condition of **ICU mortality**.

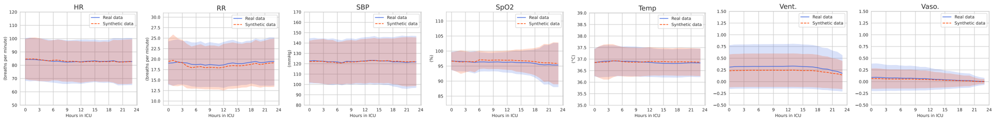

Supplementary Figure 13. Comparison of patient trajectories. The distribution of values at each timepoint (mean and standard deviation) are compared between real and synthetic patient trajectory produced by EHR-M-GAN$_{\texttt{cond}}$, under the condition of **Hospital mortality**.

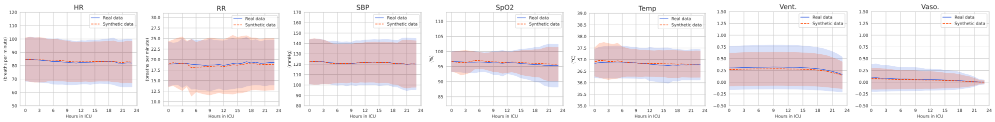

Supplementary Figure 14. Comparison of patient trajectories. The distribution of values at each timepoint (mean and standard deviation) are compared between real and synthetic patient trajectory produced by EHR-M-GAN$_{\texttt{cond}}$, under the condition of **30-day readmission**.

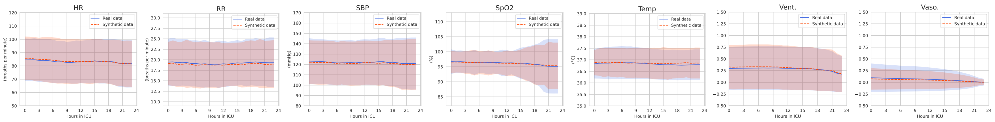

Supplementary Figure 15. Comparison of patient trajectories. The distribution of values at each timepoint (mean and standard deviation) are compared between real and synthetic patient trajectory produced by EHR-M-GAN$_{\texttt{cond}}$, under the condition of **No 30-day readmission**.

REFERENCES

1. Olof Mogren. C-rnn-gan: Continuous recurrent neural networks with adversarial training. *arXiv preprint arXiv:1611.09904*, 2016.
2. Cristóbal Esteban, Stephanie L Hyland, and Gunnar Rätsch. Real-valued (medical) time series generation with recurrent conditional gans. *arXiv preprint arXiv:1706.02633*, 2017.
3. Jinsung Yoon, Daniel Jarrett, and Mihaela Van der Schaar. Time-series generative adversarial networks. *Advances in neural information processing systems*, 32, 2019.
4. Ziqi Zhang, Chao Yan, Thomas A Lasko, Jimeng Sun, and Bradley A Malin. Synteg: a framework for temporal structured electronic health data simulation. *Journal of the American Medical Informatics Association*, 28(3):596–604, 2021.
5. Ziqi Zhang, Chao Yan, and Bradley A Malin. Keeping synthetic patients on track: feedback mechanisms to mitigate performance drift in longitudinal health data simulation. *Journal of the American Medical Informatics Association*, 29(11):1890–1898, 2022.
6. Dongha Lee, Hwanjo Yu, Xiaoqian Jiang, Deevakar Rogith, Meghana Gudala, Mubeen Tejani, Qiuchen Zhang, and Li Xiong. Generating sequential electronic health records using dual adversarial autoencoder. *Journal of the American Medical Informatics Association*, 27(9): 1411–1419, 2020.
7. Kieran Chin-Cheong, Thomas Sutter, and Julia E Vogt. Generation of heterogeneous synthetic electronic health records using gans. In *workshop on machine learning for health (ML4H) at the 33rd conference on neural information processing systems (NeurIPS 2019)*. ETH Zurich, Institute for Machine Learning, 2019.
8. Chao Yan, Ziqi Zhang, Steve Nyemba, and Bradley A Malin. Generating electronic health records with multiple data types and constraints. In *AMIA annual symposium proceedings*, volume 2020, page 1335. American Medical Informatics Association, 2020.
9. Shannon KS Kroes, Matthijs van Leeuwen, Rolf HH Groenwold, and Mart P Janssen. Generating synthetic mixed discrete-continuous health records with mixed sum-product networks. *Journal of the American Medical Informatics Association*, 2022.
10. Ziyu Wan, Bo Zhang, Dongdong Chen, Pan Zhang, Dong Chen, Jing Liao, and Fang Wen. Old photo restoration via deep latent space translation. *arXiv preprint arXiv:2009.07047*, 2020.
11. Diederik P Kingma and Max Welling. Auto-encoding variational bayes. *arXiv preprint arXiv:1312.6114*, 2013.
12. Ming-Yu Liu, Thomas Breuel, and Jan Kautz. Unsupervised image-to-image translation networks. In *Advances in neural information processing systems*, pages 700–708, 2017.
13. Ming-Yu Liu and Oncel Tuzel. Coupled generative adversarial networks. *Advances in neural information processing systems*, 29:469–477, 2016.
14. Shirly Wang, Matthew BA McDermott, Geeticka Chauhan, Marzyeh Ghassemi, Michael C Hughes, and Tristan Naumann. Mimic-extract: A data extraction, preprocessing, and representation pipeline for mimic-iii. In *Proceedings of the ACM Conference on Health, Inference, and Learning*, pages 222–235, 2020.
15. Zhengping Che, Sanjay Purushotham, Kyunghyun Cho, David Sontag, and Yan Liu. Recurrent neural networks for multivariate time series with missing values. *Scientific reports*, 8(1): 1–12, 2018.
16. Mike Wu, Marzyeh Ghassemi, Mengling Feng, Leo A Celi, Peter Szolovits, and Finale Doshi-Velez. Understanding vasopressor intervention and weaning: risk prediction in a public heterogeneous clinical time series database. *Journal of the American Medical Informatics Association*, 24(3):488–495, 2017.
17. Hugo Yèche, Rita Kuznetsova, Marc Zimmermann, Matthias Hüser, Xinrui Lyu, Martin Faltys, and Gunnar Ratsch. Hirid-icu-benchmark—a comprehensive machine learning benchmark on high-resolution icu data. 2021.
18. Alistair EW Johnson, Tom J Pollard, Lu Shen, H Lehman Li-Wei, Mengling Feng, Mohammad Ghassemi, Benjamin Moody, Peter Szolovits, Leo Anthony Celi, and Roger G Mark. Mimic-iii, a freely accessible critical care database. *Scientific data*, 3(1):1–9, 2016.
19. Shengpu Tang, Parmida Davarmanesh, Yanmeng Song, Danai Koutra, Michael W Sjoding, and Jenna Wiens. Democratizing ehr analyses with fiddle: a flexible data-driven preprocessing pipeline for structured clinical data. *Journal of the American Medical Informatics Association*, 27(12):1921–1934, 2020.
20. Tom J Pollard, Alistair EW Johnson, Jesse D Raffa, Leo A Celi, Roger G Mark, and Omar Badawi. The eicu collaborative research database, a freely available multi-center database for critical care research. *Scientific data*, 5(1):1–13, 2018.

21. Stephanie L Hyland, Martin Faltys, Matthias Hüser, Xinrui Lyu, Thomas Gumbsch, Cristóbal Esteban, Christian Bock, Max Horn, Michael Moor, Bastian Rieck, et al. Early prediction of circulatory failure in the intensive care unit using machine learning. *Nature medicine*, 26(3): 364–373, 2020.
22. Alexander Meyer, Dina Zverinski, Boris Pfahringer, Jörg Kempfert, Titus Kuehne, Simon H Sündermann, Christof Stamm, Thomas Hofmann, Volkmar Falk, and Carsten Eickhoff. Machine learning for real-time prediction of complications in critical care: a retrospective study. *The Lancet Respiratory Medicine*, 6(12):905–914, 2018.
23. Sanjay Purushotham, Chuizheng Meng, Zhengping Che, and Yan Liu. Benchmarking deep learning models on large healthcare datasets. *Journal of biomedical informatics*, 83:112–134, 2018.
24. Emma Rocheteau, Pietro Liò, and Stephanie Hyland. Temporal pointwise convolutional networks for length of stay prediction in the intensive care unit. In *Proceedings of the Conference on Health, Inference, and Learning*, pages 58–68, 2021.
25. Karol Gregor, Ivo Danihelka, Alex Graves, Danilo Rezende, and Daan Wierstra. Draw: A recurrent neural network for image generation. In *International conference on machine learning*, pages 1462–1471. PMLR, 2015.
26. Tim Salimans, Ian Goodfellow, Wojciech Zaremba, Vicki Cheung, Alec Radford, and Xi Chen. Improved techniques for training gans. *Advances in neural information processing systems*, 29, 2016.
27. Siddharth Biswal, Soumya Ghosh, Jon Duke, Bradley Malin, Walter Stewart, Cao Xiao, and Jimeng Sun. Eva: Generating longitudinal electronic health records using conditional variational autoencoders. In *Machine Learning for Healthcare Conference*, pages 260–282. PMLR, 2021.
28. Jianmin Bao, Dong Chen, Fang Wen, Houqiang Li, and Gang Hua. Cvae-gan: fine-grained image generation through asymmetric training. In *Proceedings of the IEEE international conference on computer vision*, pages 2745–2754, 2017.
29. Bin Dai and David Wipf. Diagnosing and enhancing vae models. *arXiv preprint arXiv:1903.05789*, 2019.
30. Gaurav Parmar, Dacheng Li, Kwonjoon Lee, and Zhuowen Tu. Dual contradistinctive generative autoencoder. In *Proceedings of the IEEE/CVF Conference on Computer Vision and Pattern Recognition*, pages 823–832, 2021.
