# Supplementary material for: Generating synthetic mixed-type longitudinal electronic health records for artificial intelligent applications
Source: NPJ Digit Med. 2023 May 27;6:98. doi: 10.1038/s41746-023-00834-7 (PMC10224668; doi:10.1038/s41746-023-00834-7)
Supplement: Supplementary file 2 — Reporting Summary [file 41746_2023_834_MOESM2_ESM.pdf]

nature portfolio

Corresponding author(s): Tingting Zhu

Last updated by author(s): Apr 17, 2023

Reporting Summary

Nature Portfolio wishes to improve the reproducibility of the work that we publish. This form provides structure for consistency and transparency in reporting. For further information on Nature Portfolio policies, see our Editorial Policies and the Editorial Policy Checklist.

Statistics

For all statistical analyses, confirm that the following items are present in the figure legend, table legend, main text, or Methods section.

n/a	Confirmed	
☐	☒	The exact sample size (*n*) for each experimental group/condition, given as a discrete number and unit of measurement
☐	☒	A statement on whether measurements were taken from distinct samples or whether the same sample was measured repeatedly
☐	☒	The statistical test(s) used AND whether they are one- or two-sided<br>*Only common tests should be described solely by name; describe more complex techniques in the Methods section.*
☐	☒	A description of all covariates tested
☒	☐	A description of any assumptions or corrections, such as tests of normality and adjustment for multiple comparisons
☐	☒	A full description of the statistical parameters including central tendency (e.g. means) or other basic estimates (e.g. regression coefficient) AND variation (e.g. standard deviation) or associated estimates of uncertainty (e.g. confidence intervals)
☐	☒	For null hypothesis testing, the test statistic (e.g. *F*, *t*, *r*) with confidence intervals, effect sizes, degrees of freedom and *P* value noted<br>*Give P values as exact values whenever suitable.*
☒	☐	For Bayesian analysis, information on the choice of priors and Markov chain Monte Carlo settings
☒	☐	For hierarchical and complex designs, identification of the appropriate level for tests and full reporting of outcomes
☒	☐	Estimates of effect sizes (e.g. Cohen's *d*, Pearson's *r*), indicating how they were calculated

Our web collection on statistics for biologists contains articles on many of the points above.

Software and code

Policy information about availability of computer code

Data collection	All datasets (MIMIC-III, eICU-CRD, and HiRID) are publicly available from PhysioNet, therefore we did not perform any primary data collection.
Data analysis	The source codes of this paper is available at https://github.com/jli0117/ehrMGAN. The models were built based on Python (3.7), with the deep learning framework implemented under Tensorflow (1.14.0).

For manuscripts utilizing custom algorithms or software that are central to the research but not yet described in published literature, software must be made available to editors and reviewers. We strongly encourage code deposition in a community repository (e.g. GitHub). See the Nature Portfolio guidelines for submitting code & software for further information.

Data

Policy information about availability of data

All manuscripts must include a data availability statement. This statement should provide the following information, where applicable:

- Accession codes, unique identifiers, or web links for publicly available datasets
- A description of any restrictions on data availability
- For clinical datasets or third party data, please ensure that the statement adheres to our policy

All datasets are publicly available from PhysioNet: MIMIC-III (https://physionet.org/content/mimiciii/1.4/); eICU-CRD (https://physionet.org/content/eicu-crd/2.0/); HiRID (https://physionet.org/content/hirid/1.1.1/). Further code repositories for data preprocessing (such as concept extractions via PostgreSQL, and benchmark model implementations) can be accessed from the corresponding Github links: MIMIC-III (https://github.com/MIT-LCP/mimic-code); eICU-CRD (https://github.com/MIT-LCP/eicu-code); HiRID (https://github.com/ratschlab/HIRID-ICU-Benchmark).

Field-specific reporting

Please select the one below that is the best fit for your research. If you are not sure, read the appropriate sections before making your selection.

☒ Life sciences ☐ Behavioural & social sciences ☐ Ecological, evolutionary & environmental sciences

For a reference copy of the document with all sections, see nature.com/documents/nr-reporting-summary-flat.pdf

Life sciences study design

All studies must disclose on these points even when the disclosure is negative.

Sample size	All available data were downloaded from three publicly available datasets, and were filtered based on the exclusion criteria (see below). Sample sizes after applying the exclusion criteria are: MIMIC-III dataset = 28,344; eICU-CRD dataset = 99,015; HiRID dataset = 14,129. Details of the underlying population distributions for the corresponding labels can be found in main manuscript.
Data exclusions	The data exclusion criteria were pre-established for all three critical care databases while in line with the previous literature: (1) Only the first known ICU admission of the patient is selected; (2) Patient has to be an adult at the time of ICU admission; (3) The duration of the patients' ICU stay is at least 12 hours and less than 10 days. In-depth details can be found in the attached supplementary materials.
Replication	All experiments were performed independently at least 8 times, and the corresponding statistics (mean and standard deviation) over these results are reported. Experimental protocol is described in the main article (see Fig. 5 for experimental setup), and detailed implementations are also provided in the Github repository (https://github.com/jli0117/ehrMGAN).
Randomization	Patients were split into training (70%) and testing (30%) sets randomly by the stratified sampling method (https://scikit-learn.org/stable/modules/generated/sklearn.model_selection.train_test_split.html). Experiments were also conducted across random seeds during repetitions (i.e., random weight initializations and random data batches). During downstream task evaluation, the percentage for augmentation with randomly selected samples were adopted at three different levels (10%, 25% and 50%) to supplement the training set (see subsection for Downstream tasks).
Blinding	Blinding is not applicable in our case as this study did not compare experimental groups.

Reporting for specific materials, systems and methods

We require information from authors about some types of materials, experimental systems and methods used in many studies. Here, indicate whether each material, system or method listed is relevant to your study. If you are not sure if a list item applies to your research, read the appropriate section before selecting a response.

Materials & experimental systems

n/a	Involved in the study	
☒	☐	Antibodies
☒	☐	Eukaryotic cell lines
☒	☐	Palaeontology and archaeology
☒	☐	Animals and other organisms
☒	☐	Human research participants
☒	☐	Clinical data
☒	☐	Dual use research of concern

Methods

n/a	Involved in the study	
☒	☐	ChIP-seq
☒	☐	Flow cytometry
☒	☐	MRI-based neuroimaging
